# Real-world cost-effectiveness of pan-genotypic Sofosbuvir-Velpatasvir combination versus genotype dependent directly acting anti-viral drugs for treatment of hepatitis C patients in the universal coverage scheme of Punjab state in India

**DOI:** 10.1371/journal.pone.0221769

**Published:** 2019-08-29

**Authors:** Yashika Chugh, Radha Krishan Dhiman, Madhumita Premkumar, Shankar Prinja, Gagandeep Singh Grover, Pankaj Bahuguna

**Affiliations:** 1 Department of Community Medicine and School of Public Health, Post Graduate Institute of Medical Education and Research, Chandigarh, India; 2 Department of Hepatology, Post Graduate Institute of Medical Education and Research, Chandigarh, India; 3 Mukh Mantri Punjab Hepatitis C Relief Fund (MMPHCRF), Punjab Government, Punjab, India; 4 Technical Resource Group, National Viral Hepatitis Control Program (NVHCP), Government of India, Ministry of Health and Family Welfare, New Delhi, India; 5 Hepatitis C Virus Infection, Directorate of Health and Family Welfare, Punjab, India; BITS Pilani, INDIA

## Abstract

**Background:**

We undertook this study to assess the incremental cost per quality adjusted life year (QALY) gained with the use of pan-genotypic sofosbuvir (SOF) + velpatasvir (VEL) for HCV patients, as compared to the current treatment regimen under the universal free treatment scheme in Punjab state.

**Methodology:**

A Markov model depicting natural history of HCV was developed to simulate the progression of disease. Three scenarios were compared: I (Current Regimen)—use of SOF + daclatasvir (DCV) for non-cirrhotic patients and ledipasvir (LDV) or DCV with SOF ± ribavirin (RBV) according to the genotype for cirrhotic patients; II—use of SOF + DCV for non-cirrhotic patients and use of SOF+VEL for compensated cirrhotic patients (with RBV in decompensated cirrhosis patients) and III—use of SOF+VEL for both non-cirrhotic and compensated cirrhotic patients (with RBV in decompensated cirrhosis patients). The lifetime costs, life-years and QALYs were assessed for each scenario, using a societal perspective. All the future costs and health outcomes were discounted at an annual rate of 3%. Finally, the incremental cost per QALY gained was computed for each of scenario II and III, as compared to scenario I and for scenario III as compared to II. In addition, we evaluated the lifetime costs and QALYs among HCV patients for each of scenario I, II and III against the counterfactual of ‘no universal free treatment scheme’ scenario which involves patients purchasing care in routine setting of from public and private sector.

**Results:**

Each of the scenarios I, II and III dominate over the no universal free treatment scheme scenario, i.e. have greater QALYs and lesser costs. The use of SOF+VEL only for cirrhotic patients (scenario II) increases QALYs by 0.28 (0.03 to 0.71) per person, and decreases the cost by ₹ 5,946 (₹ 1,198 to ₹ 14,174) per patient, when compared to scenario I. Compared to scenario I, scenario III leads to an increase in QALYs by 0.44 (0.14 to 1.01) per person, and is cost-neutral. While the mean cost difference between scenario III and I is—₹ 2,676 per patient, it ranges from a cost saving of ₹ 14,835 to incurring an extra cost of ₹ 3,456 per patient. For scenario III as compared II, QALYs increase by 0.16 (0.03 to 0.36) per person as well as costs by ₹ 3,086 per patient which ranges from a cost saving of ₹ 1,264 to incurring an extra cost of ₹ 6,344. Shift to scenario II and III increases the program budget by 5.5% and 60% respectively.

**Conclusion:**

Overall, the use of SOF+VEL is highly recommended for the treatment of HCV infection. In comparison to the current practice (scenario I), scenario II is a dominant option. Scenario III is cost-effective as compared to scenario II at a threshold of one-time GDP per capita. If budget is an important constraint, velpatasvir should be given to HCV infected cirrhotic patients. However, if no budget constraint, universal use of velpatasvir for HCV treatment is recommended.

## Introduction

Viral Hepatitis has become the 7^th^ leading cause of death worldwide [1]. More than 90% of the deaths and disability due to viral hepatitis can be attributed to hepatitis C virus (HCV) and hepatitis B virus (HBV) infections, the rest being attributed to hepatitis A and E [2]. The number of persons infected with HCV in India has been reported to be 6–12 million [3]. While anti-HCV prevalence in India ranges between 0.9% and 1.9% [4–6], it has been reported to be as high as 3.6% in north Indian state of Punjab [7–8].

Poor access to diagnosis and treatment for HCV is a significant factor contributing to high mortality. In 2015, out of 71 million people estimated to be living with chronic HCV infection globally, only 20% were diagnosed. Among those diagnosed, only 7.4% receive the treatment. Similarly, only 9% of those on treatment received the recent directly acting antiviral (DAA) based regimens [9]. This can be attributed to high cost of DAA based treatment. Moreover, there are financial and geographic barriers to accessing diagnostic facilities for genotype testing which is required prior to prescribing treatment.

The standard regimen for HCV treatment was earlier based on pegylated interferon (PEG-INF) and ribavirin (RBV), which had low sustained virologic response (SVR) as well as serious adverse events [10]. With the availability of DAA, the treatment of hepatitis C has become much more efficacious and safe, the only barrier being the high cost of the drugs. Some Indian states like Punjab, Haryana and Manipur have taken state-level measures to improve access to HCV treatment with DAAs [3]. For instance, the Punjab government launched the “*Mukh Mantri Punjab Hepatitis C Relief Fund* (MMPHCRF)” for free universal treatment of HCV patients with DAAs and thus improve access to treatment. This has been possible due to the availability of generic DAAs which have significantly lesser cost as compared to the branded DAAs, at comparable efficacy [11]. The MMPHCRF scheme was launched in 2016 and uses the existing infrastructure of 22 secondary level district hospitals and 3 tertiary level medical colleges in Punjab to deliver HCV infection related treatment and care [12]. Diagnostic services which are not available in these facilities were out-sourced from the private sector. Further, the National Viral Hepatitis Control Programme (NVHCP) has also been launched in India which envisages provision of free diagnostic and treatment facilities to tackle the growing burden of HCV. The national guidelines for the diagnosis and management [13] of HCV have been prepared which are in accordance with the World Health Organisation (WHO) management guidelines [14].

The current DAA being used are effective but are genotype dependent which in turn is dependent on limited availability of diagnostic facilities at district and sub-district level. Non-cirrhotic patients do-not require genotyping prior to initiation of treatment. Also, the current treatment regimen for non-cirrhotic patients which is daclatasvir (DCV) based, has high SVR rates. But for cirrhotic patients, genotyping is required as genotype 3 patients are treated with DCV and non-genotype 3 patients with Ledipasvir (LDV) and also SVR rates are low. A possible solution to this may be the introduction of newer but costlier pan-genotypic DAA, i.e. velpatasvir (VEL) in combination with sofosbuvir (SOF). This regimen has high effectiveness with SVR rates being as high as 99%. Since it is pan-genotypic, there is no need for genotype testing of cirrhotic patients [15–16]. Though WHO recommends the use of VEL with SOF ± ribavirin (RBV), the Punjab Government constituted a Task Force to evaluate the cost-effectiveness of introducing Velpatasvir for treatment of HCV patients in the universal MMPHCRF scheme to analyse the same in local context. Since VEL is expensive as compared to LDV/DCV (which give high SVR rates for non-cirrhotic patients) and resources are limited, it is worthwhile to make an informed policy decision whether to give VEL to both cirrhotic and non-cirrhotic patients or to only cirrhotic patients. In this context, we undertook this study to assess the incremental cost per quality adjusted life year (QALY) gained for 2 possible scenarios of introduction of VEL–a combination of SOF/VEL when used only for cirrhotic HCV patients alone; or for both non-cirrhotic and cirrhotic patients, as compared to the current treatment regimen SOF in combination with LDV and DCV.

Barring a few states which have initiated efforts for increasing the coverage of HCV treatment through provision of free treatment, the overall coverage of HCV treatment remains low in the other states. Therefore, in addition to above comparisons, all the 3 treatment scenarios (I, II and III) described above were compared against a scenario of no universal treatment scheme (no-treatment/do-nothing situation) in which we assumed that only 10% of the population was receiving the newer DAA (LDV/DCV) based treatment as per current coverage rates of treatment.

## Methods

### Overview of analysis

**Fig 1:**
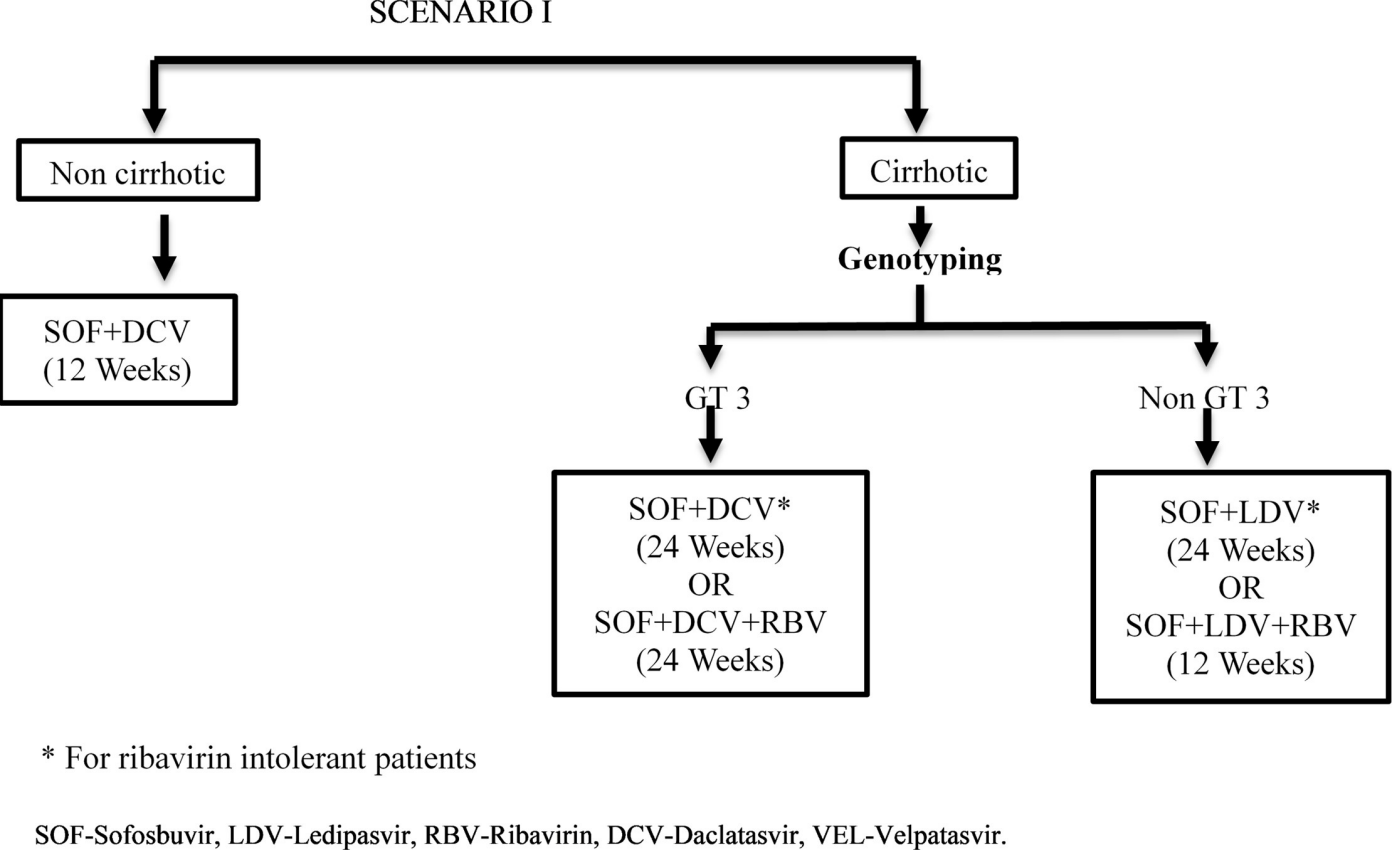
Treatment algorithm for Hepatitis C virus patients under “MukhMantri Punjab Hepatitis C Relief Fund (MMPHCRF)” in Punjab state, India–Routine care.

**Fig 2:**
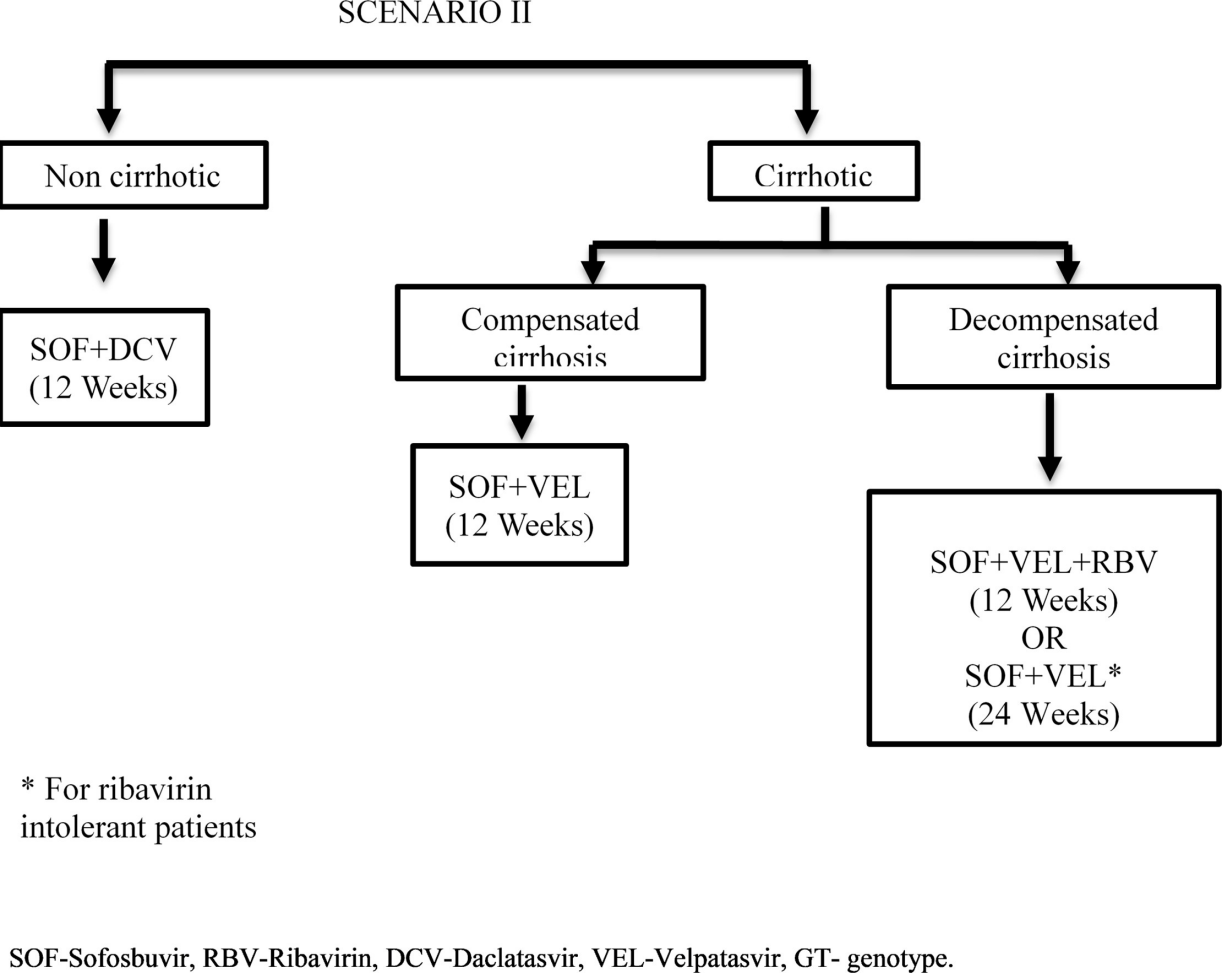
Treatment algorithm for Hepatitis C virus patients under “National Viral Hepatitis Control Program, India–Intervention (Scenario II).

**Fig 3:**
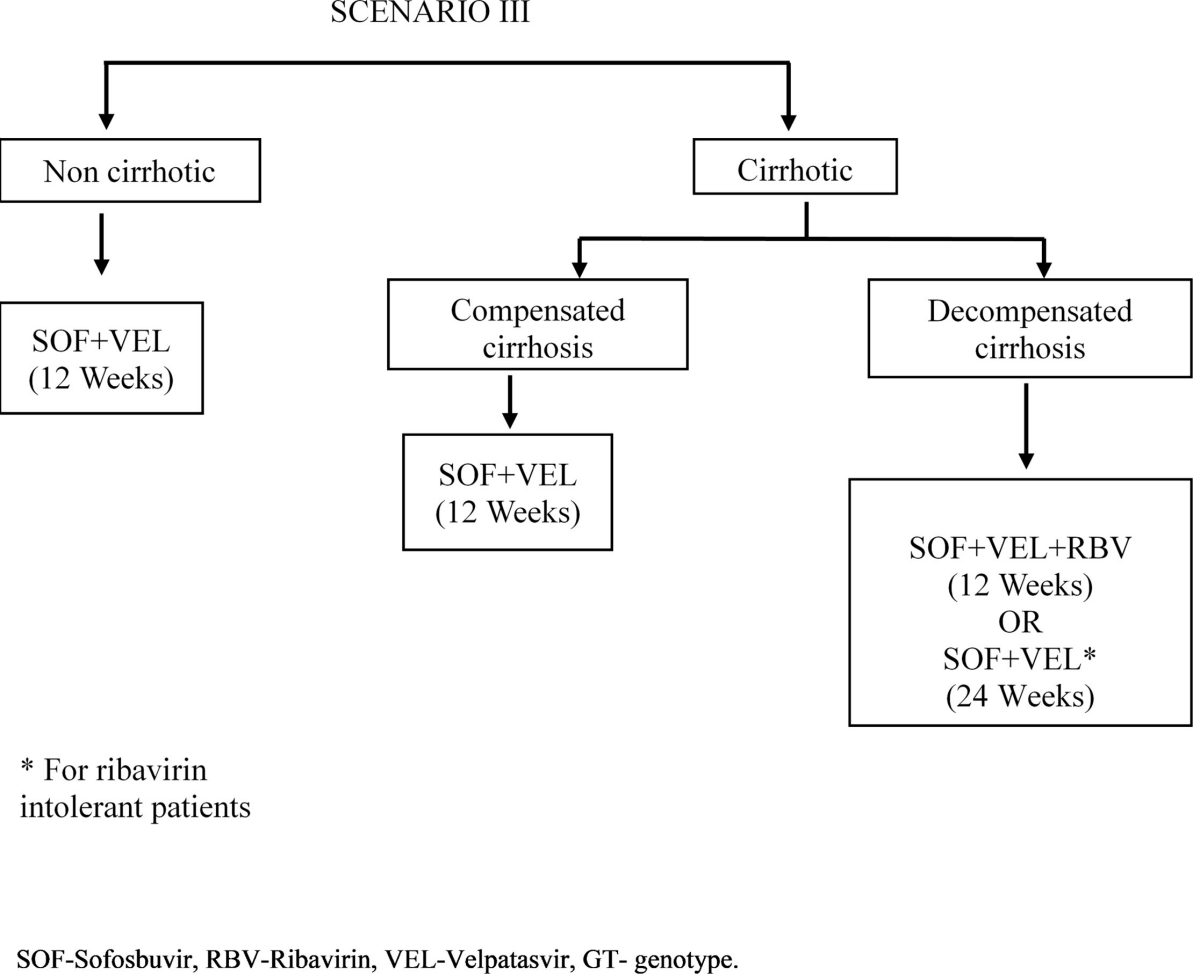
Proposed treatment algorithm for hepatitis C virus patients under “National Viral Hepatitis Control Program, India–Intervention (Scenario III).

We modelled the lifetime costs and outcomes for HCV patients from Punjab in 4 different scenarios–‘do nothing’ / no-universal treatment scheme, scenario I which is the current treatment algorithm (Fig 1), scenario II where SOF-VEL is used for treatment of cirrhotic patients while remaining treatment algorithm remains same as scenario I (Fig 2), and scenario III where SOF-VEL is used to treat all HCV patients (Fig 3). Outcomes are valued in terms of the number of HCV deaths, life years and QALYs. Future costs and consequences are discounted at 3% based on standard international guidelines as well as for consistency with other Indian economic evaluations [17–20]. These methodological principles are also consistent with the Indian reference case for conducting economic evaluations which has recently been published by the health technology agency in India [20]. Finally, we compared the costs and consequences of each of the 3 treatment scenarios (I, II and III) with do-nothing. Secondly, we compared scenario II and III with scenario I and also scenario III with II. The standard guidelines for conducting and reporting an economic evaluation survey (CHEERS) were adhered to and details are available as S1 File.

#### No treatment/ do-nothing scenario

We model this scenario to determine the costs and outcomes if no universal free treatment scheme to improve access to treatment is introduced. Under this scenario, as per the reported coverage rates elsewhere in India [9,21], we assume that only 10% of the HCV infected cohort receives the treatment and rest progress to higher stages as per the natural progression of HCV infection. The treatment regimens for these 10% patients are similar to the MMPHCRF current strategy (scenario I) which is explained below. The remaining patients who do not receive DAA based treatment incur cost of supportive management including the management of associated complications. The drugs and diagnostic prices for this scenario are as per the market prices of these drugs and not as per MMPHCRF rates. This is because the significantly lower cost of drugs is due to the operating bulk procurement mechanisms under this scheme.

#### Routine care scenario (scenario I)

The present treatment algorithm for HCV patients in Punjab is described in Fig 1. All non-cirrhotic patients receive SOF+DCV for 12 weeks. For the patients with cirrhosis, genotype 3 patients receive SOF+DCV with or without RBV for 12 or 24 weeks. Non-genotype 3 patients receive SOF+LDV with or without RBV for 12 or 24 weeks. All the patients with cirrhosis have to undergo genotyping prior to the initiation of treatment.

90% of the total infected cohort was assumed to be treated in this scenario so as to account for 10% increase in coverage of treatment with the introduction of VEL in scenario II and III for which we assumed 100% coverage.

#### Intervention scenarios (II and III)

The routine care scenario I was compared with two alternative scenarios. In the first (Scenario II, Fig 2), all non-cirrhotic patients were assumed to receive SOF+DCV for 12 weeks which is same as in routine scenario. All the patients with compensated cirrhosis receive SOF+VEL for 12 weeks. For decompensated cirrhosis, the RBV tolerant patients receive SOF+VEL+RBV for 12 weeks whereas the RBV intolerant receive SOF+VEL for 24 weeks. Thus, the genotyping procedure was discontinued for cirrhotic patients due to the introduction of VEL.

In the second alternative intervention strategy (Scenario III, Fig 3), all non-cirrhotic and compensated cirrhosis patients receive SOF+VEL for 12 weeks. The regimen for decompensated cirrhosis patients is the same as described above in the scenario II. These two intervention scenarios were also compared and both these intervention scenarios assume 100% treatment.

### Model structure

**Fig 4:**
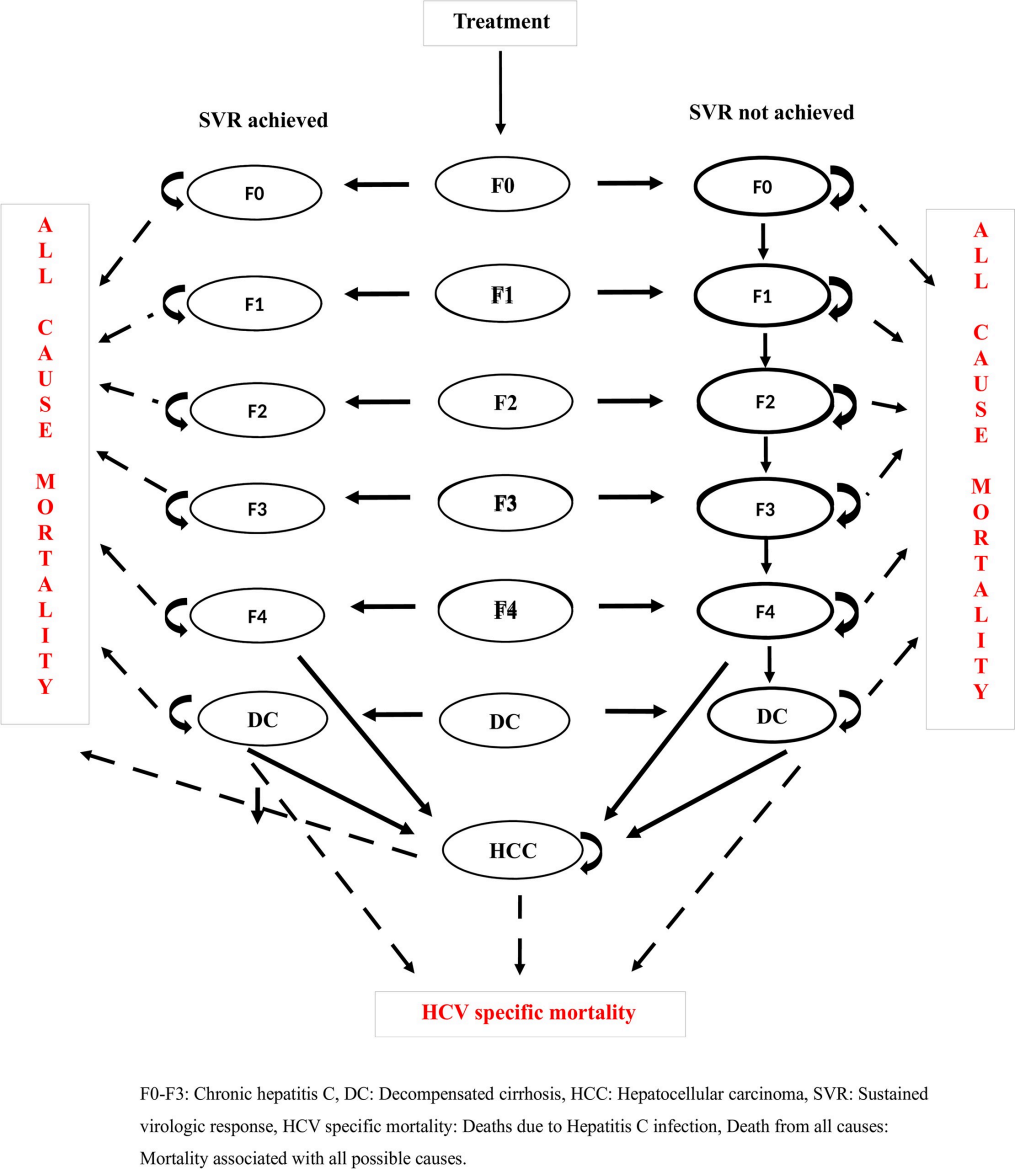
Markov model for progression of Hepatitis C virus (HCV) infection.

A mathematical Markov model, depicting natural history of HCV (Fig 4) was prepared in Microsoft Excel to simulate the progression of disease, estimate the costs, health outcomes of each intervention scenario (II and III) compared with the routine care (scenario I) and for each of these scenarios (I, II and III) compared with the do-nothing / no universal treatment scheme scenario.

A Markov model with 9 transition states including 1) F0, no fibrosis; 2) F1, portal fibrosis without septa; 3) F2, portal fibrosis with few septa; 4) F3, numerous septa without cirrhosis; 5) F4, cirrhosis; 6) decompensated cirrhosis; and 7) hepatocellular carcinoma (HCC) was developed [22]. Two absorbing states included 8) HCV related mortality and 9) all-cause mortality (Fig 4). Liver transplant was not included in the model pertaining to pertaining to its very low coverage in real-wold setting. According to the Census of India (2011), the population of Punjab was approximately 28 million, and prevalence of anti-HCV in Punjab is estimated to be 3.6% and viremic patients (HCV RNA positive) is 2.6%, with an estimated burden of around 728,000 viremic hepatitis C patients [7–8,23]. All viremic patients in Punjab were assumed to be given treatment and thus the cohort size was assumed to be 728,000 [7–8,23]. Median age for HCV infection, based on the analysis of the MMPHCRF patient data, was estimated to be 41 years (Table 1). Cirrhotic patients were categorised into genotype 3 (70%) or non-genotype 3 (30%) and whether there is tolerance to ribavirin (90%) or not (10%). Subsequent to treatment, the patients were divided into those who achieve SVR and those who do not. The patients who do not achieve SVR continue to progress to subsequent stages as per the natural progression of the disease. The patients in stages F0-F3 who achieve SVR, do not progress further and remain in the same stage. For the patients with cirrhosis who achieve SVR, continue to progress to subsequent stages but at a slower rate. Death due to HCV was assumed to occur from decompensated cirrhosis and HCC states. Based on the total duration of treatment as well as subsequent follow-up investigations, a cycle length of 1 year was considered appropriate along with a life-time study horizon so as to comprehensively capture all the intended costs and expected benefits of the intervention.

**Table 1:** Demographic, epidemiologic, effectiveness and cost-related parameters.

Parameters			Base value	Lower limit	Upper limit	Distribution	Source
Proportion of viraemic population			0.026	0.026	0.026	Uniform	7
Stage-wise distribution at diagnosis		F0	0.325	0.30875	0.34125	Uniform	Authors’ analysis of MMPHCRF data
		F1	0.325	0.30875	0.34125	Uniform	
		F2	0.1	0.095	0.105	Uniform	
		F3	0.1	0.095	0.105	Uniform	
		F4	0.12	0.114	0.126	Uniform	
		DC	0.03	0.0285	0.0315	Uniform	
Proportion of population having genotype 3			0.7	0.63	0.77	Uniform	Authors’ analysis of MMPHCRF data
Proportion of population that is RBV tolerant			0.9	0.81	0.99	Uniform	
						
Quality of life weights		F0—F3	0.63	0.57	0.70	Beta	
		F4	0.56	0.51	0.61	Beta	
		DC	0.44	0.38	0.49	Beta	Primary data collection and analysis
		HCC	0.44	0.38	0.49	Beta	
		F0-F3 (post SVR)	1	0.83	1	Beta	
						
Discount rate			0.03	0.02	0.08	Uniform	
Transition probabilities		F0 to F1	0.177	0.104	0.13	Beta	33
		F1 to F2	0.085	0.075	0.096	Beta	33
		F2 to F3	0.12	0.109	0.133	Beta	33
		F3 to F4	0.116	0.104	0.129	Beta	33
		F4 to DC	0.035	0.027	0.043	Beta	34
		F4 to DC (post SVR)	0.002	0.0001	0.005	Beta	34
		F4 to HCC	0.024	0.018	0.031	Beta	34
		F4 to HCC (post SVR)	0.005	0.001	0.009	Beta	34
		DC to HCC	0.068	0.03	0.083	Beta	34
		DC to HCC (post SVR)	0.03	0.0225	0.0375	Beta	34
Probability of dying due to HCV		F0 –F4	0	0	0	Uniform	Expert opinion
		DC	0.216	0.162	0.27	Uniform	38–40
		HCC	0.411	0.31	0.51	Uniform	38–40
SVR rates (%)							
*Non-Cirrhotic*		SOF+DCV (12 weeks)	74	66.5	73.5	Uniform	Authors’ analysis of MMPHCRF data for LDV/DCV based regimens, Authors’ estimation based on trials (15–16,34–35) for VEL based regimens.
		SOF+VEL (12 weeks)	86	81.7	90.3	Uniform	
*Compensated cirrhosis*	GT 3	SOF+DCV (24 weeks)	66.8	63.4	70.1	Uniform	
		SOF+DCV+RBV (24 weeks)	66.8	63.4	70.1	Uniform	
		SOF+VEL (12 weeks)	84	79.8	88.2	Uniform	
	Non-GT 3	SOF+LDV (24 weeks)	66.8	63.4	70.1	Uniform	
		SOF+LDV+RBV (12 weeks)	66.8	63.4	70.1	Uniform	
		SOF+VEL (12 weeks)	86	81.7	90.3	Uniform	
*Decompensated cirrhosis*	GT 3	SOF+DCV (24 weeks)	66.8	63.4	70.1	Uniform	
		SOF+DCV+RBV (24 weeks)	66.8	63.4	70.1	Uniform	
		SOF+VEL+RBV (12 weeks)	84	79.8	88.2	Uniform	
	Non-GT 3	SOF+LDV (24 weeks)	66.8	63.4	70.1	Uniform	
		SOF+LDV+RBV (12 weeks)	66.8	63.4	70.1	Uniform	
		SOF+VEL (24 weeks)	81	76.95	85.05	Uniform	
		SOF+VEL+RBV (12 weeks)	84	79.8	88.2	Uniform	
*Treatment coverage in do-nothing scenario (%)*			10	5	20	Uniform	9,21
*Increase in coverage when shifting from genotype dependent to pan genotypic regimens (%)*			10	0	25	Uniform	42
Drug costs-12 weeks (₹)							
*MMPHCRF rates*		SOF+DCV	4509	3607.2	4509	Gamma	11
		SOF+DCV+RBV	8223	6578.4	8223	Gamma	11
		SOF+LDV	9576	7660.8	9576	Gamma	11
		SOF+LDV+RBV	13290	10632	13290	Gamma	11
		SOF+VEL	13104	10483.2	13104	Gamma	11
		SOF+VEL+RBV	16818	13454.4	16818	Gamma	11
*Market prices*		SOF+DCV	52500	4509	52500	Gamma	
		SOF+DCV+RBV	55293	8223	55293	Gamma	
		SOF+LDV	55500	9576	55500	Gamma	
		SOF+LDV+RBV	58293	13290	58293	Gamma	
Cost of diagnostic tests (₹)							
*MMPHCRF rates*		ELISA	50	40	75	Gamma	11
		HCV-RNA	880	704	1320	Gamma	11
		Routine tests (CBC, LFT, Creatinine)	500	400	750	Gamma	11
		Cirrhosis evaluation (Fibro-scan)	350	280	525	Gamma	11
		Genotyping	895	716	1342.5	Gamma	11
*Market prices*		ELISA	100	70	130	Gamma	
		HCV-RNA	5000	3500	6500	Gamma	
		Routine tests (CBC, LFT, Creatinine)	700	490	910	Gamma	
		Cirrhosis evaluation (Fibro-scan)	1650	1320	2475	Gamma	
		Genotyping	5500	3850	7150	Gamma	
Proportion of patients utilising public sector for OPD		Secondary	1	0.91	1	Beta	Authors’ analysis of MMPHCRF data
		Tertiary	0.1	0.09	0.11	Beta	Authors’ analysis of MMPHCRF data
Proportion of patients utilising public sector for OPD		Secondary	0.06	0	0.154	Beta	Authors’ analysis of MMPHCRF data
		Tertiary	0.94	0.846	1	Beta	
Cost per OPD consultation (₹)		Primary	1686.3	1180.41	2192.19	Gamma	25–26
		Secondary	1734	1213.8	2254.2	Gamma	25–26
		Tertiary	2024	1416.8	2631.2	Gamma	24,27
Cost per patient hospitalisation (₹)		Primary	6347.1	4442.97	8251.23	Gamma	25–26
		Secondary	7597	5317.9	9876.1	Gamma	25–26
		Tertiary	18693	13085.1	24300.9	Gamma	24,27

HCV-Hepatitis C virus, DC-Decompensated cirrhosis, HCC-Hepatocellular carcinoma, SOF-Sofosbuvir, LDV-Ledipasvir, RBV-Ribavirin, DCV-Daclatasvir, VEL-Velpatasvir, GT-genotype, OPD- Out-patient Department, IPD- In-patient Department, ₹: Indian rupee.

### Costing

Costs were analysed from a societal perspective (including both the health system and out-of-pocket expenditures), and comprised of cost of out-patient (OPD) consultation for diagnostic and therapeutic purpose, in-patient care for hospitalisation in advanced stages as well as cost of specific procedures, such as endoscopy, fibro-scan, ultrasound, ascitic tap which are required for the management of complications. Data pertaining to number of OPD contacts required, proportion of patients that require hospitalisation and number of hospitalisations per patient per year for each stage of HCV infection was based on authors’ clinical experience and the practice under the MMPHCRF scheme. For a patient in F0-F4 stages, 3 OPD contacts are required on an average per year. Only 5% patients were assumed to require hospitalisation, with an average 2 hospitalization events per patient per year. Similarly, a patient in decompensated cirrhosis and HCC stage requires 12 OPD contacts per year. It was assumed that 80% patients of decompensated cirrhosis and 60% patients of HCC would require hospitalisation, with an average of 6 hospitalisations for DC patients and 2 hospitalisations for HCC patients per year. Data for the costs of hospitalisation was obtained from a study undertaken in a large tertiary care hospital of north India to assess the cost of intensive care treatment of liver disorders [24]. Average costs for primary, secondary and tertiary OPD were reported to be ₹ 1686 (US $ 24), ₹ 1734 (US $ 24.7) and ₹ 2024 (US $ 28.9) respectively. Similarly, these costs for IPD were ₹ 6347 (US $ 90), ₹ 7597 (US $ 108) and ₹ 18693 (US $ 267) [24–26]. The tertiary sector costs for IPD included both the cost of medicine ward [27] as well as the intensive care unit (ICU) costs. The costs of diagnostic tests were estimated from the provider payment rates of Punjab government contracted laboratories [12]. The cost of genotyping (₹ 895, US $ 12.7) was also as per the rates prescribed in MMPHCRF scheme which has decreased by 70% from the initial cost as the scheme evolved [12]. Similarly, the cost of DAAs (ranging from ₹ 4000–17000, US $ 57–242) were as per the procurement prices in Punjab [11]. Generic drugs were supplied by Indian pharmaceutical companies NATCO Pharma Limited, (Hyderabad, India) and Zydus Cadila (Ahmedabad, India), which are licensed by the originator company Gilead Sciences, Inc. to manufacture the generic drugs DCV (60 mg), SOF (400 mg) and a fixed dose combination of SOF (400 mg) + LDV (90mg), at FDA approved facilities. Central Drugs Standard Control Organization, Directorate General of Health Sciences, FDA Bhawan, New Delhi granted permission to manufacture these new drugs formulations for the treatment of CHC patients. Their costs have significantly decreased due to the bulk procurement mechanisms [28]. For the do-nothing scenario, the drug and diagnostic costs were according to the market prices as in the absence of MMPHCRF scheme, there were no such operating bulk procurement mechanisms and thus the high cost (ranging from ₹ 50,000–120,000, US $ 714–1,714). All the costs which have been included are listed in Table 1. All costs were also reported in USD at a currency exchange rate of 1 USD = 70.1 INR [29].

Out of pocket (OOP) expenditure of the patient was derived based on analysis of unit level data from National Sample Survey 71^st^ round, conducted in during January-June 2014 [30]. This data comprises of a large household survey covering a sample of 65,932 households. OOP expenditure was estimated for outpatient and inpatient care among those who reported viral hepatitis.

Finally, we also obtained data on the existing budget of the MMPHCRF–which comprises on allocation for procurement of drugs, purchasing of diagnostic services as well as installation of a tele-consultation system. Further, we evaluated the change in budgetary allocation for the scheme which would be required for scenario II and III respectively.

### Valuation of consequences

The effect of intervention reported in terms of SVR was modelled to assess progression to more severe health states and finally in terms of survival. The health-related quality of life was measured using Euro-QOL five dimensions questionnaire (EQ-5D-5L) which was administered to a total of 230 patients who were being treated for chronic HCV infection (40 patients), compensated cirrhosis (40 patients), decompensated cirrhosis and hepato-cellular carcinoma (150 patients). These patients were either hospitalized or visited the outpatient department (OPD) for treatment in a tertiary care hospital of north India and were recruited via consecutive sampling method. Out of the total, these 150 patients of decompensated cirrhosis and hepato-cellular carcinoma were those admitted in intensive care unit (ICU) or high dependency unit (HDU). These patients were interviewed at the time of admission and were also followed up to 6 months for post-treatment evaluation. Due to deaths that occurred in this duration, the post hospitalisation QOL data was collected for remaining 120 patients. Finally, the QOL reported at 6 months was considered for the model as it represented the more stable QOL in the given health state. The index QOL score was estimated using the tariff values from Thailand for EQ-5D-5L [31]. In view of the absence of a value set for Indian population, the draft guidelines for health technology assessment (HTA) by the HTA Board also recommend the use of Thailand’s tariff values till Indian value set is generated [20].

The quality of life among non-cirrhotic patients who achieve SVR, was assumed to be equivalent to the general population [32]. For the cirrhotic and hepato-cellular carcinoma patients, the quality of life score was similar as in the respective advanced stages. The patients not achieving SVR continued to progress to subsequent stages according to the natural progression rates.

The parameters related to progression of the disease (with and without achieving SVR) were obtained from published literature [33–34]. The data pertaining to SVR rates achieved for the regimens in current practice (Scenario I) in Punjab was based on authors’ analysis of MMPHCRF data which included a large sample of 48,088 patients. The pooled SVR rates for all HCV patients who completed treatment, was found to be 91.2%. However, SVR rate falls to 69.5% if analysed as intention to treat. In a public health set-up, such difference is bound to occur and is attributed to loss to follow-up, treatment interruptions, and treatment failures that happened in the duration of treatment which account for 20–25% of the total recruited patients as per the MMPHCRF data. Thus, we assumed a pooled SVR rate of 69.5% for scenario I (SOF-LDV/DCV). For VEL based regimens, though trial data is available but true SVR rates as in a public health set-up have not been reported yet. SVR rates range from 85 to 99% for SOF+VEL based regimens according to the published trial data [15–16.^35^–^36^] which is similar to the SVR rates achieved in Indian trial settings [37]. Therefore, we used these SVR rates for VEL based regimens (scenario II and III) from available trial data. However, to maintain comparability across all the scenarios and to report a pragmatic analysis, we assumed a 20–25% reduction in SVR rates for VEL based regimens (as reported in trial setting) so as to account for the decrease in SVR rates when intervention is delivered in a public health set-up. In order to further validate this assumption, we undertook a univariate sensitivity analysis to assess the threshold SVR rate, which is required for scenarios II and III to remain cost-saving as compared to scenario I.

Annual HCV related mortality from decompensated cirrhosis and hepato-cellular carcinoma was assumed to be 21.6% and 41.1% respectively [38–40]. The transition probabilities considered have been listed in Table 1. The age-wise all-cause mortality was obtained from the Sample Registration System report for Punjab state [41]. Though the MMPHCRF provides free drugs and diagnostic facilities, the cost of genotype testing has to be borne by the patients themselves and thus inability to access this facility acts as a barrier for patients to get the required treatment. Therefore, an increase in coverage of treatment by 10% was assumed as we move from genotype dependent regimens (scenario I) to pan-genotypic VEL based regimens (scenario II and III). This is consistent with assumption made in a previous analysis from Indian perspective [42].

### Sensitivity analysis

A probabilistic sensitivity analysis (PSA) was undertaken to analyse the effect of joint parameter uncertainty. Monte Carlo method was used and the results were simulated 999 times. The epidemiological parameters, including the stage-wise distribution at the time of diagnosis and SVR rates was varied by 5% for both upper and lower limits, the genotype distribution and distribution of cohort according to RBV tolerance was varied by 10% for both upper and lower limits. All the drugs and diagnostic test costs were varied by 20% on the lower side only. The current prices, as available by MMPHCRF, are the negotiated prices as a result of bulk procurement which might further decrease but any further increase in the costs is unlikely. Assuming the same for do-nothing scenario, the drugs and diagnostic costs were varied only on the lower side, the limit being the present MMPHCRF negotiated prices. Uniform distribution was used for the treatment effectiveness parameters, gamma distribution for all the cost parameters and beta distribution was used for the transition probabilities and QOL weights. Based on the PSA, probability for scenario II and III as compared to I, scenario III as compared to II as well as for scenario I as compared to do-nothing to be cost-effective at varying willingness to pay thresholds (WTP) was estimated. While the WHO recommends a range of 1–3 times per capita gross-domestic product (GDP) for considering an intervention as cost-effective [43], much lower thresholds for India have been suggested based on a recent work [44]. Based on a review of the existing cost effectiveness studies done in India [19], we have used India’s per capita GDP to comment on cost effectiveness of scenario II and III.

We undertook a scenario analysis for assessing the cost effectiveness of scenario II and III as compared to scenario I, wherein we did not assume any increase in coverage of treatment due to the introduction of pan-genotypic VEL. Thus, this scenario analysis primarily evaluated the cost effectiveness of alternative treatment strategies with VEL which could be attributed to differences in effectiveness of drugs.

Another scenario analysis was undertaken where-in the drug prices for all the scenarios were corresponding to the present market prices. This scenario analysis evaluated the impact of coverage of treatment on the cost-effectiveness of different scenarios as compared to do-nothing. A univariate sensitivity analysis was also conducted to assess the impact of various parameters individually that are likely to affect the cost-effectiveness of the intervention scenarios.

## Results

### Costs

In the do-nothing scenario, where only 10% of the HCV infected patients are assumed to receive treatment, a lifetime cost of ₹ 106,822 (US $ 1,524) is incurred per patient. Delivering care to HCV patients in the current routine scenario (I) incurs a lifetime societal cost of ₹ 65,306 (US $ 932) per patient. Adoption of scenario II, i.e, giving SOF+VEL with or without RBV to cirrhotic patients and SOF+DCV to non-cirrhotic patients, costs ₹ 59,674 (US $ 851) per patient. Despite VEL being a costly drug as compared to DCV and LDV, the Scenario II saves ₹ 5,946 (US $ 85) per patient when compared with the routine scenario. The cost savings per patient range from ₹ 1,198 (US $ 17) to ₹ 14,174 (US $ 202). Despite an increase in cost of drugs, scenario II is cost saving due to significant reduction in cost of managing complications of severe stages, as well as reduction in cost of genotyping.

The scenario III, i.e, giving SOF+VEL with or without RBV, costs ₹ 62,477 (US $ 891) per patient. When compared to the scenario I, it is cost neutral, i.e. an average reduction of ₹ 2,676 (US $ 38) per patient, which varies from a cost-saving of ₹ 14,835 (US $ 212) to incurring an extra cost of ₹ 3,456 (US $ 49) per patient. When scenario III is compared to scenario II, average cost per patient increase by ₹ 3086 (US $ 44), which varies from a cost-saving of ₹ 1264 (US $ 18) to incurring an extra cost of ₹ 6344 (US $ 90) (Table 2).

**Table 2:** Cost and effects of treating HCV patients.

**Do-nothing**	
Life years per patient	10.98
QALY per patient	6.93
Cost per patient (₹, US $)	106,822 (US $ 1523)
**Scenario I**	
Life years per patient	11.31
QALY per patient	8.7
Cost per patient (₹, US $)	65,306 (US $ 931)
**Scenario II**	
Life years per patient	11.38
QALY per patient	9
Cost per patient (₹, US $)	59,674 (US $ 851)
**Scenario III**	
Life years per patient	11.4
QALY per patient	9.18
Cost per patient (₹, US $)	62,477 (US $ 891)
**Incremental gains**	
***Scenario II-I***	
Life years gained	0.07 (0.02–0.17)
QALYs gained	0.28 (0.03–0.71)
Cost difference (₹)	-5,946 (-14,174 to– 1,198)
***Scenario III-I***	
Life years gained	0.09 (0.03–0.22)
QALYs gained	0.44(0.14–1.01)
Cost difference (₹)	-2,676 (-14,835 to 3456)
***Scenario III-II***	
Life years gained	0.01 (0.003–0.04)
QALYs gained	0.16 (0.03–0.36)
Cost difference (₹)	3086 (-1,246 to 6,344)

HCV: Hepatitis C virus, QALY: Quality adjusted life year, ₹: Indian rupee, US $: United States Dollar.

In terms of budgetary allocations compared to the current outlay under the MMPHCRF (scenario I), scenario II and III would lead to an overall increase of 5.5% and 60% respectively.

### Valuation of consequences

The life years lived by HCV patients in do-nothing, scenario I, II and III were 10.98, 11.31, 11.38 and 11.40 per patient respectively. Similarly, the QALYs lived per patient in do-nothing, scenario I, II and III were 6.93, 8.70, 9.00 and 9.18 respectively. As compared to the routine scenario (I), there was a gain of 0.07 (0.02–0.17) life years and 0.28 (0.03–0.71) QALY per patient in scenario II. For scenario III, this gain was 0.09 (0.03–0.22) life years and 0.44 (0.14–1.01) QALY per patient when compared to the routine scenario (I). When scenario III is compared with II, there is a gain of 0.01 (0.003–0.04) life years and 0.16 (0.03–0.36) QALYs per patient. However, there is statistically insignificant difference between the QALYs gained for the above three comparisons. When all the scenarios (I, II and III) were compared with do-nothing situation, there was a gain of 1.75, 2.04 and 2.22 QALYs per patient respectively (Table 2).

Table 3 gives intermediate HCV related health outcomes as well as cost break-up for drugs, diagnostics, genotyping and management costs for all the scenarios.

**Table 3:** Hepatitis C related health outcomes and cost breakup for a cohort of 600,000 patients.

**HCV infection related health outcomes (in thousands)**				
**Cases**	**Do-nothing**	**Scenario I**	**Scenario II**	**Scenario III**
F4	2,065	1,564	1,547	1,469
DC	256	149	130	122
HCC	137	67	54	50
**Cost breakup in millions (**₹**, US $)**				
Drugs and diagnostics	9,969 (142)	8,363 (119)	8,823 (126)	13,373 (191)
Management cost	90,997 (1,298)	47,568 (679)	41,690 (595)	37,613 (537)
Genotyping	495 (7.1)	77 (1.1)	0 (0)	0 (0)
Total cost	101,461 (1,447)	56,008 (799)	50,513 (721)	50,986 (727)

HCV: Hepatitis C virus, F4: Compensated cirrhosis, DC: Decompensated cirrhosis, HCC: Hepatocellular carcinoma, ₹: Indian rupee, US $: United States Dollar

### Cost-effectiveness

Each treatment scenario (scenario I, II and III) dominates over no treatment, i.e. has better health outcome and is cost saving (Table 4). Secondly, among different treatment options, scenario II (SOF-VEL for cirrhotic patients alone) dominates over scenario I, i.e. better health outcomes (higher QALYs and life years) and saves cost. Scenario III was also found to have significantly positive gain in QALYs than scenario I. However, when the incremental gains of scenario II and III (relative to scenario I) are compared, there is no statistically significant difference in QALYs. In addition, scenario III is cost-neutral as compared to scenario I (no significant difference), while scenario II is cost saving (significantly lesser cost) than scenario I. Similar findings were demonstrated when scenario III was compared with II. Though the QALY gains are positive but there is no statistically significant difference when compared to incremental gains for scenario II and III as compared to I. Also, scenario III is cost-neutral when compared to II. Thirdly, if the assumption of an increase in coverage of treatment due to the introduction of pan-genotypic VEL is not considered, even then also scenario II dominates over I whereas Scenario III is cost-neutral when compared to I and II. Fourthly, when we consider market prices for drugs instead of MMPHCRF prices, even then then the median estimate reflects cost-savings of ₹ 6242 (US $ 89) with incremental gain of 0.28 QALYs per patient for scenario II compared to I. This further demonstrates that the dominance is not only due to reduced drug prices but also due to coverage of treatment. The results for comparison of all scenarios with drug prices as market prices have been given as S2 File.

**Table 4:** Cost-effectiveness of alternative treatment scenarios for HCV patients in Punjab state.

		Total costs per patient (₹, US $)	Total QALYs per patient	ICER/QALY gained compared to do-nothing (₹, US $)	ICER/QALY gained compared to current scenario I (₹, US $)	ICER/QALY gained compared to scenario II (₹, US $)
Do-nothing		106,822 (US $ 15240	6.93			
Current scenario I	Coverage: Scenario I– 90% Scenario II & III—100%	65,306 (US $ 932)	8.70	Dominant ₹ -23,562 (-36,123 to -15,181) US $ -336 (-515 to -216)		
	Without assuming the increase in coverage due to VEL (Coverage in all three scenarios same– 100%)	59,136 (US $ 844)	8.74	Dominant ₹ -22,539 (-35,888 to -14,702) US $ -321 (-511 to -210)		
Scenario II	Coverage: Scenario I– 90% Scenario II & III—100%	59,674 (US $ 851)	9.00		Dominant ₹ -21,506 (-40,231 to -13,183) US $ -307 (-574 to -188)	
	Without assuming the increase in coverage due to VEL (Coverage in all three scenarios same– 100%)	58,473 (US $ 834)	8.77	Dominant ₹ -22,818 (-36,220 to -14,938) US $ -326 (-517 to -213)	Dominant ₹ -54,611 (-106,505 to -22,653) US $ -779 (-1519 to -323)	
Scenario III	Coverage: Scenario I– 90% Scenario II & III—100%	62,477 (US $ 891)	9.18		More effective but Cost-neutral ₹ -5,633 (-19,248 to 20,595) US $ -80 (-275 to 294)	More effective but Cost-neutral ₹ 18,258 (-4538 to 153,839) US $ 260 (-64.7 to 2195) (when scenario III is compared to II, there is no role of the coverage assumption as both scenarios as VEL based and assume 100% coverage)
	Without assuming the increase in coverage due to VEL (Coverage in all three scenarios same– 100%)	61,379 (US $ 876)	8.93	Dominant ₹ -19,543 (-31,785 to -12,195) US $ - 279 (-453 to -174)	More effective but Cost-neutral ₹ -11,567 (-7,462 to 108,537) US $ 165 (-106 to 1548)	

HCV: Hepatitis C virus, QALY: Quality adjusted life year, ₹: Indian rupee, ICER: Incremental cost-effectiveness ratio, VEL: Velpatasvir.

### Sensitivity analysis

**Fig 5:**
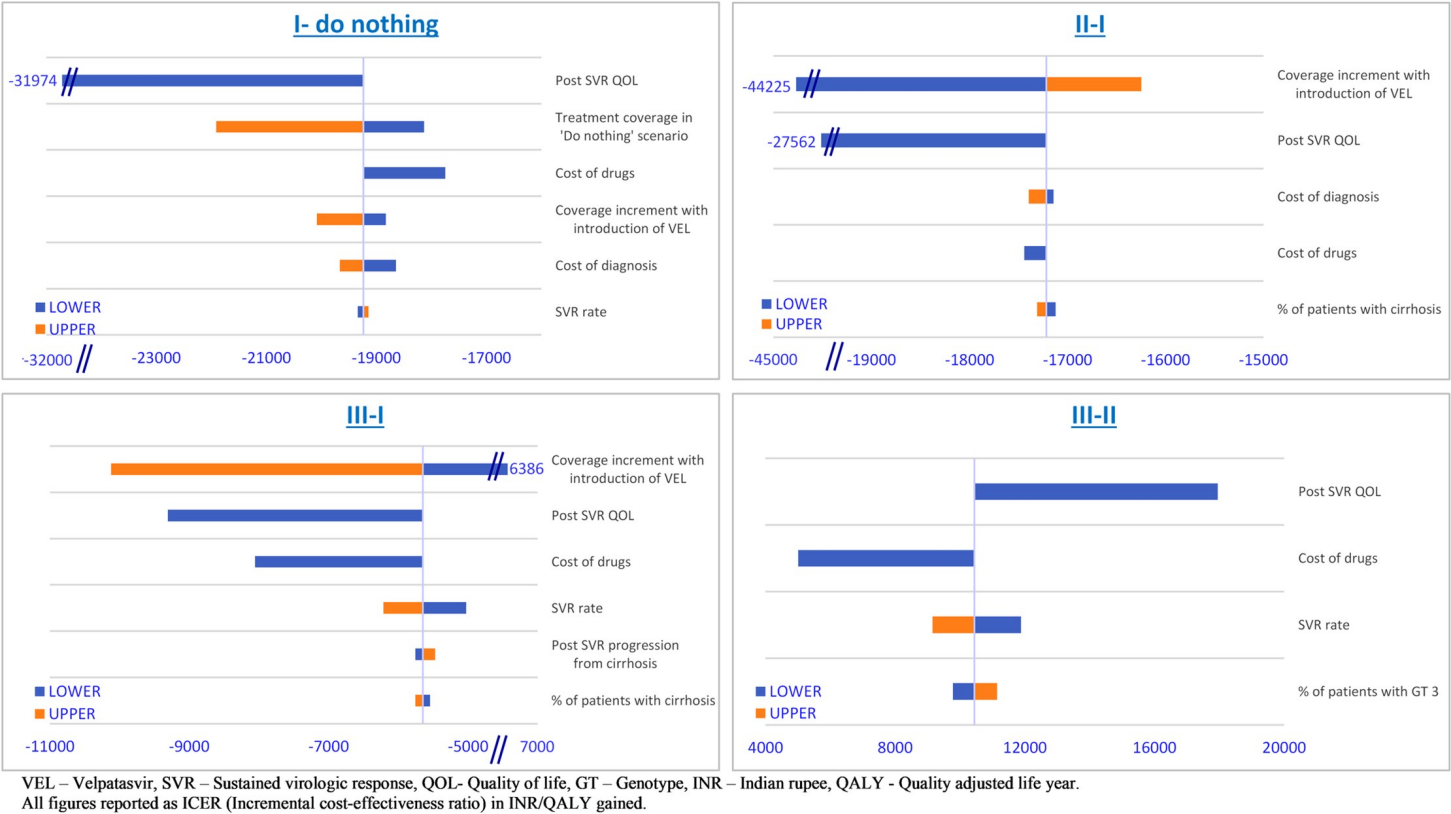
Tornado plot showing the effect of individual parameters on incremental cost-effectiveness ratio (ICER) for different scenarios.

A univariate sensitivity analysis was conducted to estimate the impact of individual parameters on the ICER. Parameters pertaining to treatment efficacy, genotype distributions, drugs and diagnostic costs, coverage increase due to introduction of VEL were analysed. The analysis is most sensitive to the increase in coverage with introduction of VEL, post SVR QOL for non-cirrhotic patients and the cost of drugs. Fig 5 represents tornado diagrams for scenario I compared to do-nothing, scenario II & III compared to I and scenario III compared to II.

Threshold for the efficacy of VEL up-to which the interventions remain cost saving/effective was also estimated. For the SVR rate as low as 65% for VEL, scenario II still remains cost-saving as compared to scenario I. For scenario III-I, it remains cost-saving up-to SVR rate of 73% and remains cost-effective afterwards for efficacy of VEL as low as 65% at an ICER of ₹ 52,314 (US $ 746/QALY gained). The efficacy for VEL was not decreased beyond 65% for threshold analysis as this would be a value lower than what exists for SVR rate for LDV/DCV which is practically inappropriate.

**Fig 6:**
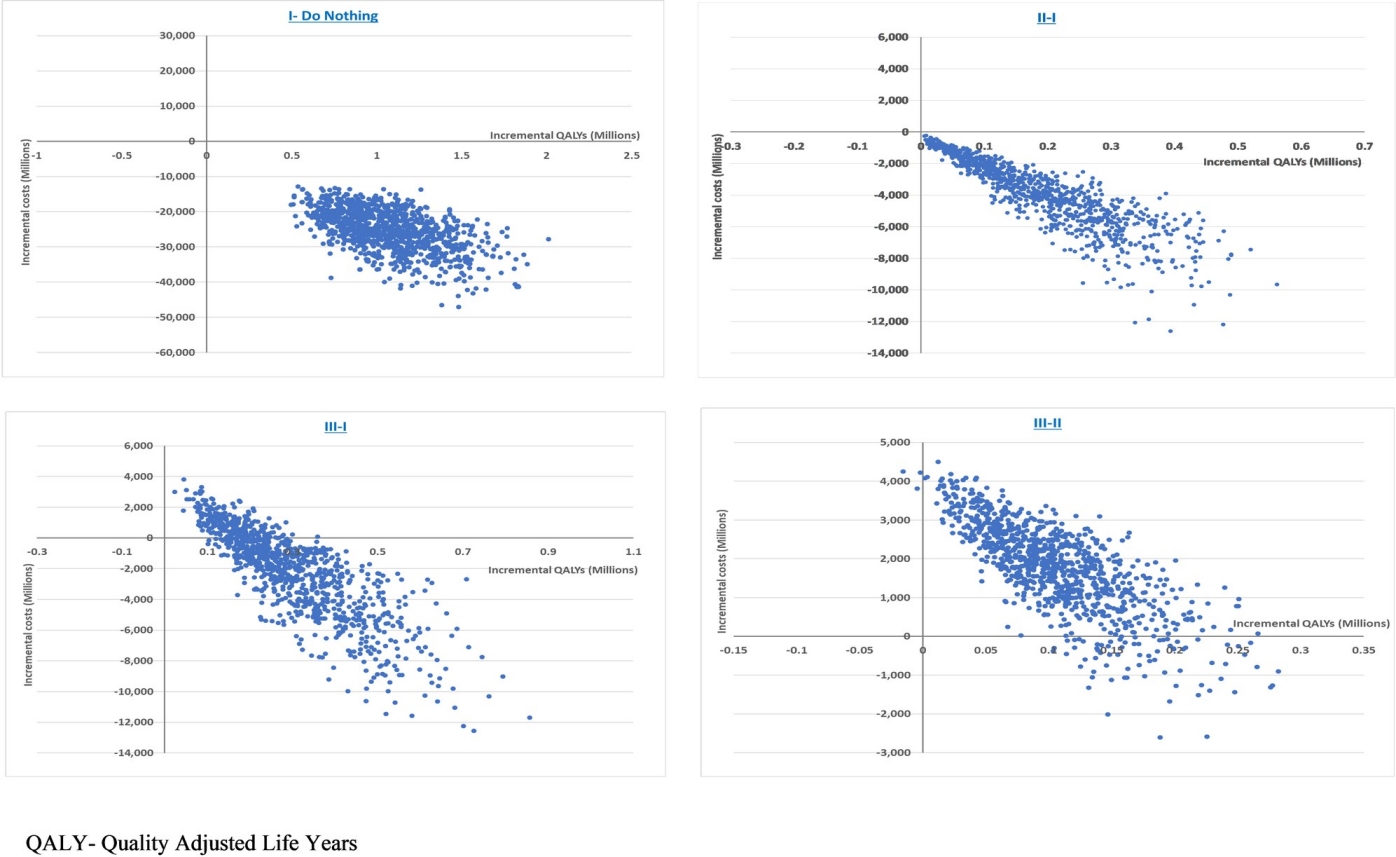
Cost-effectiveness planes for different treatment scenarios compared.

**Fig 7:**
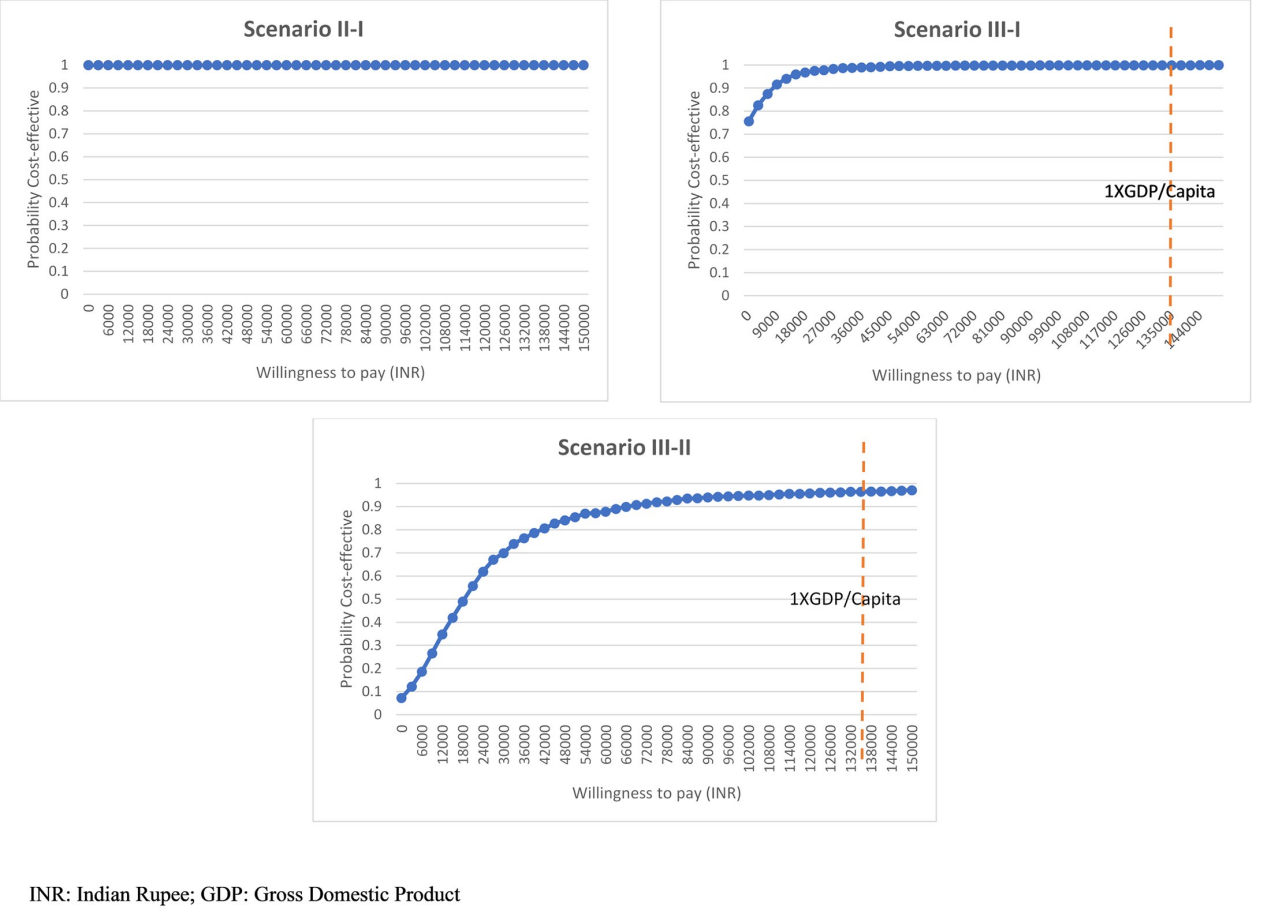
Cost-effectiveness Acceptability Curves for different treatment scenarios compared.

Even after accounting for the impact of joint uncertainty in parameter values using PSA, scenario II had a 100% probability of being a dominant scenario as compared to scenario I. As compared to scenario II, scenario III has a 96% probability of being cost-effective at a willingness to pay of per-capita GDP, i.e, ₹ 135,966 (US $ 1939) The cost-effectiveness planes and acceptability curves for different scenarios generated as a result of Monte Carlo simulations have been given in Figs 6 and 7 respectively.

## Discussion

In the context of continuously rising costs of health care, cost-effectiveness analysis serves as an important tool for rational decision making and guiding policy actions. The present economic evaluation was undertaken to assess the incremental cost per QALY gained when using SOF+VEL for only cirrhotic patients (scenario II) or for both non-cirrhotic and cirrhotic patients (scenario III) as compared to SOF+LDV/DCV (scenario I). Our findings conclude that the use of SOF/VEL for treating Hepatitis C is very cost-effective. When compared to current practice (scenario I), Scenario II is cost-saving. Scaling up the coverage of VEL to all HCV patients, i.e. Scenario III is cost-effective in comparison to Scenario II at a threshold of one-time GDP per capita. From a pure health maximization point of view, universal application of VEL for all HCV patients is recommended. However, budget impact of any change in strategy is also an important feasibility consideration in most low-and- middle income countries. The services under MMPHCRF scheme are horizontally integrated within the health system which is funded through a supply-side financing mechanism. From health system perspective, implementation of scenario III will increase the current health system budgetary outlay by 60%, whereas scenario II leads to an increase in budgetary allocation by 5.5%. It is important to highlight that while the efficiency analysis is undertaken from economic costing using societal perspective, budgetary impact is evaluated from a financial costing using health system perspective. Thus, with a pure health maximization point of view, universal use of VEL (Scenario III) should be the preferred strategy. Whereas in resource limited settings, use of VEL for only cirrhotic patients (Scenario II) should be adopted both on grounds of value for money and budget constraint.

The fact that SOF+VEL for cirrhotic HCV patients is a cost-saving dominant strategy can be attributed to decrease in overall treatment duration, reduction in the management costs for complications of late stages, and discontinuation of genotyping procedure for cirrhotic patients. For patients with compensated cirrhosis, the duration of treatment with SOF+VEL is 12 weeks. On the contrary, the duration of treatment in scenario I is 24 weeks along with addition of RBV (except for RBV tolerant non-genotype 3 patients where duration is 12 weeks). The improved health outcomes are attributable to improved SVR rates which leads to reduced progression to complicated late stages as well as the increase in coverage of treatment due to introduction of this pan-genotypic drug which obviates the need for limited genotyping facilities.

At a threshold of one-time GDP per capita, universal use of SOF/VEL (Scenario III) is cost-effective. The increase in drug cost is offset by decrease in patient management costs in VEL based treatment scenario, with 85% of these being non-cirrhotic who stop progressing and continue to be in the same stage post achieving the SVR and thus lesser number of patients progress to higher stages of decompensated cirrhosis and hepato-cellular carcinoma. When comparing scenario II to routine scenario I, there is a decrease in number of patients who advance to DC and HCC by 37%. This percentage rises to 53% when scenario III is compared to I. For a patient of decompensated cirrhosis/hepato-cellular carcinoma, average management cost per year ranges from ₹ 60,000–100,000 (US $ 857–1428). With the introduction of universal use of VEL, this cost can be averted for almost half of the patients who would have otherwise been in these advanced stages.

Prior to the availability of LDV and DCV, the standard of care for treatment of chronic HCV was the use of PEG-INF+RBV. The SVR rates achieved were very low as compared to the DAAs that are available now. The newer DAA i.e. VEL has demonstrated further increase in efficacy but the price is higher than LDV and DCV. Making these drugs affordable for the treatment of millions of people has been possible due to availability of generic versions of these drugs which have revolutionised the treatment of HCV. Secondly, the recent reforms in drug procurement systems in India–which involves centralized procurement and decentralized distribution, provides monopsonistic power to the Government agencies which procure drugs [28,45–46] who are now able to negotiate and reduce market prices of drugs from manufacturers. This was also evident in the case of Punjab state where the Department of Health was able to procure DAAs at a significantly reduced price–₹ 4000–17000 (US $ 57–242) for a 12-week course[12]. In India, the market price of generic SOF+VEL ranges from ₹ 50,000–60,000 (US $ 714–857) for a 12-week course [47] which is almost 4 times the price at which the Punjab government is procuring the same combination and is providing it free to the patients. This clearly demonstrates that the scope for potential price negotiations significantly increases with effective implementation of such universal schemes. Thirdly, the rates of viral load testing (₹ 880, US $ 12.5) and genotyping (₹ 895, US $ 12.7) under the Punjab government scheme are significantly low–again as a result of strategic purchasing [12].

A previous model-based cost effectiveness analysis of VEL use among HCV patients reported that universal use of VEL is cost-saving in Indian context[42]. Our findings in terms of health consequences are similar, and more realistic, to what has been reported in the previous study if we assume an increase in the coverage of treatment with the introduction of VEL. Very few studies have been done to assess the cost-effectiveness of VEL for low and middle-income countries (LMIC). Moreover, it is difficult to compare various studies due to methodological differences. A study based in US was done to evaluate the cost-utility of HCV treatment with elbasvir/grazoprevir (EBR/GZR) regimens compared to SOF/LDV, ombitasvir/paritaprevir/ritonavir + dasabuvir ± ribavirin (3D ± RBV), and SOF/VEL in genotype-1 infection. The use of SOF/VEL was reported to be cost-effective when compared with SOF-DCV ± RBV and SOF/LDV for both treatment naïve and experienced patients. Universal use of SOF/VEL yields QALYs in the range of 13–15 per patient as compared to 9.18 QALYs per patient as per our analysis [48]. This difference can be attributed to the difference in the quality of life weights used for the analysis which are significantly lower for Indian population as compared to the US population. Secondly, while the mean age for onset of HCV infection was assumed to be 20 years in US, we assumed a median age for HCV infection to be 41 years.

The major strength of our paper is use of a biologically plausible model which is fitted with local data on disease epidemiology, effectiveness of DAAs, cost of treatment, and in terms of the pragmatic policy question being explored. In the available published evidence regarding cost-effectiveness of generic DAA for developing countries, there is a lack of empirically estimated data regarding actual stage-wise distribution of HCV patients, hospitalisation as well drugs and diagnostic costs and quality of life of HCV patients [42,49]. In our analysis, the data for stage-wise distribution, genotype distribution, cost of drugs and diagnostics was available from local data of Punjab state as collected under MMPHCRF. Secondly, the SVR rates used for LDV/DCV based regimens are based on analysis of MMPHCRF data for 48,088 HCV patients which was done using standard methods along with adjusting for loss to follow-up, drop outs, treatment failures and deaths. For SOF/VEL, actual effectiveness data in a public health setting was not available and therefore efficacy data from trials was used. There are practical challenges in evaluating the impact of the intervention when data is from different sources. Though real-world analysis of effectiveness is best demonstrated in a public health setting but the existing evidence for SOF/VEL is trial based. One of the problems for comparing efficacy from trials and effectiveness in a public health set-up is the controlled nature in which the treatment is administered. Besides this, difference in population characteristics, compliance, management of ancillary conditions can also contribute to better results in a trial setting. In view of this, the data from trial was the only source of efficacy of the treatment which was adjusted, as described in the methods section, to account for loss to follow-up, treatment interruptions and failures as well as deaths. The assumptions for the latter were made from MMPHCRF data.

The univariate sensitivity analysis done to assess the threshold for the efficacy of VEL up-to which the interventions remain cost saving/effective further validates this assumption. The quality of life data as well as cost estimates of hospitalisation for HCV patients were again based on primary data collected from a local setting in north India. All estimates of health system cost were derived from studies conducted in health facilities of 3 north Indian states [24–27]. Similarly, data on out-of-pocket expenditure was derived based on analysis of a nationally representative household survey [30]. Further, uncertainty in parameter values was assessed in a robust sensitivity analysis.

We found a median survival of 20 years for the HCV patients. Existing published literature suggests that the median survival for compensated and decompensated cirrhosis patients is over 12 years and 2–5 years respectively, while in the survival for chronic HCV stages who have achieved SVR is comparable to that of general population [32,40,50]. Assuming a median age of onset of 41 years and a life expectancy of 65 years in India, the median survival for those in chronic HCV stages would be nearly 24 years. Considering that nearly 85% of the total HCV patients in Punjab present in early chronic HCV stages, and high rates of SVR with DAAs, our modelled median survival for the overall cohort appears plausible.

Standard recommendations suggest incorporating a sub-group analysis in economic evaluation of any health intervention. Our study comprises of a large cohort of 48,088 patients which is so far the largest sample to be analysed in a public health set-up which has ever been done in India. The distribution of HCV parameters in terms of clinical characteristics like genotype distribution and cirrhosis status in our study is not different from what is reported in other studies [5,42,51–52]. Therefore, a sub-group analysis was not done.

We do acknowledge certain limitations of the present study. The data regarding the efficacy of VEL was taken from clinical trials conducted outside India. In rest of India, there is limited published evidence for treatment efficacy of VEL but reports similar SVR rates as in trials conducted outside India. However, for the purpose of comparability with scenario I which considers SVR rates in routine care setting, we assumed a 20–25% reduction in SVR rates of VEL to account for treatment failures and interruptions. Assessment of effectiveness of VEL based treatment regimens in routine health system setting would be an important area of research to be undertaken in future.

Secondly, we did not consider DAA experienced patients in our analysis. However, the proportion of such patients was negligible in Punjab (less than 1%). Thirdly, the patients who did not achieve SVR were not considered for re-treatment as per the design of the Punjab scheme. The treatment of DAA-experienced (LDV and DCV) patients also requires further highly effective drugs like VEL.

Fourthly, while we have included health system and OOP expenditure in our cost analysis, we did not consider productivity losses. However, there is a debate whether the productivity losses should be included at all, as it has also been argued that it is somewhat reflected in the valuation of consequences when trade-off techniques are used to elicit quality of life [53]. Moreover, there are methodological as well as parameter uncertainties which are likely to be introduced when productivity losses are accounted for.

## Conclusion

Our findings show that the treating HCV patients is cost saving and results in QALY gains. This implies that all schemes which aim at universal health coverage should include the treatment of HCV in their benefit package. This is significantly important in Indian context, which has recently announced the *Ayushman Bharat* Prime Minister’s *Jan Aarogya Yojana*, which aims to provide a health care cover of ₹500,000 (US $ 7,142)) per year for a family [54]. Among the different treatment options, use of SOF/VEL for cirrhotic patients (Scenario II) is recommended in resource limited settings with high consideration to budget. However, universal use of SOF/VEL (Scenario III) for HCV treatment is a preferred strategy to maximize population health. Large-scale procurement is also likely to lower the drug prices in future. Health-care delivery through such efficient platforms like MMPHCRF in Punjab state should be replicated across India, which would help in tackling the growing burden of HCV.

## Supporting information

S1 File CHEERS checklist. (DOC)

S2 File Scenario analysis at market prices. (XLSX)

S3 File Data pertaining to utility scores calculated for different stages of HCV infection. (XLSX)
