# Supplementary material for: Real-world cost-effectiveness of pan-genotypic Sofosbuvir-Velpatasvir combination versus genotype dependent directly acting anti-viral drugs for treatment of hepatitis C patients in the universal coverage scheme of Punjab state in India
Source: PLoS One. 2019 Aug 29;14(8):e0221769. doi: 10.1371/journal.pone.0221769 (PMC6715223; doi:10.1371/journal.pone.0221769)
Supplement: S1 File — (DOC) [file pone.0221769.s001.DOC]

**Appendix S1: CHEERS checklist—Items to include when reporting economic evaluations of health interventions**

| **Section/item** | **Item No** | **Recommendation** | **Reported on page No/ line No** |
| --- | --- | --- | --- |
| **Title and abstract** | | | |
| Title | 1 | Identify the study as an economic evaluation or use more specific terms such as “cost-effectiveness analysis”, and describe the interventions compared. | Page 1, line 1 to 3 |
| Abstract | 2 | Provide a structured summary of objectives, perspective, setting, methods (including study design and inputs), results (including base case and uncertainty analyses), and conclusions. | Page 2, line 46 to 85 |
| **Introduction** | | | |
| Background and objectives | 3 | Provide an explicit statement of the broader context for the study. | Page 3, line 93 to 99;  Page 4, line 117 to 126 |
|  |  | Present the study question and its relevance for health policy or practice decisions. | Page 4, line 126 to 134;  Page 5, line 135 to 143 |
| **Methods** | | | |
| Target population and subgroups | 4 | Describe characteristics of the base case population and subgroups analysed, including why they were chosen. | Page 8, line 211 to 218 |
| Setting and location | 5 | State relevant aspects of the system(s) in which the decision(s) need(s) to be made. | Page 3, line 102 to 109;  Page 4, line 110 to 116;  Page 4, line 126 to 132;  Page 8, line 211 to 218 |
| Study perspective | 6 | Describe the perspective of the study and relate this to the costs being evaluated. | Page 11, line 235 to 236;  Page 13, line 237 to 242 |
| Comparators | 7 | Describe the interventions or strategies being compared and state why they were chosen. | Page 4, line 129 to 134;  Page 5, line 135 to 143;  Page 6, line 163 to 179;  Page 7, line 180 to 196 |
| Time horizon | 8 | State the time horizon(s) over which costs and consequences are being evaluated and say why appropriate. | Page 8, line 224 to 225;  Page 9, line 226 to 227 |
| Discount rate | 9 | Report the choice of discount rate(s) used for costs and outcomes and say why appropriate. | Page 5, line 152 to 156; |
| Choice of health outcomes | 10 | Describe what outcomes were used as the measure(s) of benefit in the evaluation and their relevance for the type of analysis performed. | Page 13, line 281 to 285; |
| Measurement of effectiveness | 11a | *Single study-based estimates:*Describe fully the design features of the single effectiveness study and why the single study was a sufficient source of clinical effectiveness data. | Page 14, line 304 to 310;  Page 15, line 311 to 322 |
|  | 11b | *Synthesis-based estimates*: Describe fully the methods used for identification of included studies and synthesis of clinical effectiveness data. |  |
| Measurement and valuation of preference-based outcomes | 12 | If applicable, describe the population and methods used to elicit preferences for outcomes. | Page 13, line 282 to 285;  Page 14, line 286 to 302 |
| Estimating resources and costs | 13a | *Single study-based economic evaluation:* Describe approaches used to estimate resource use associated with the alternative interventions. Describe primary or secondary research methods for valuing each resource item in terms of its unit cost. Describe any adjustments made to approximate to opportunity costs. | Page 11, line 235 to 236;  Page 12, line 237 to 261;  Page 13, line 262 to 275 |
|  | 13b | *Model-based economic evaluation:*Describe approaches and data sources used to estimate resource use associated with model health states. Describe primary or secondary research methods for valuing each resource item in terms of its unit cost. Describe any adjustments made to approximate to opportunity costs. |  |
| Currency, price date, and conversion | 14 | Report the dates of the estimated resource quantities and unit costs. Describe methods for adjusting estimated unit costs to the year of reported costs if necessary. Describe methods for converting costs into a common currency base and the exchange rate. | Page 13, line 269 to 270 |
| Choice of model | 15 | Describe and give reasons for the specific type of decision-analytical model used. Providing a figure to show model structure is strongly recommended. | Page 7, line 199 to 202;  Page 8, line 203 to 211  Fig 4 |
| Assumptions | 16 | Describe all structural or other assumptions underpinning the decision-analytical model. | Page 7, line 199 to 202;  Page 8, line 203 to 211;  Page 8, line 216 to 225;  Page 15, line 326 to 332 |
| Analytical methods | 17 | Describe all analytical methods supporting the evaluation. This could include methods for dealing with skewed, missing, or censored data; extrapolation methods; methods for pooling data; approaches to validate or make adjustments (such as half cycle corrections) to a model; and methods for handling population heterogeneity and uncertainty. | Page 14, line 304 to 310;  Page 15, line 311 to 322  Page 16, line 334 to 357;  Page 17, line 358 to 363 |
| **Results** | | | |
| Study parameters | 18 | Report the values, ranges, references, and, if used, probability distributions for all parameters. Report reasons or sources for distributions used to represent uncertainty where appropriate. Providing a table to show the input values is strongly recommended. | Table 1;  Page 16, line 343 to 345 |
| Incremental costs and outcomes | 19 | For each intervention, report mean values for the main categories of estimated costs and outcomes of interest, as well as mean differences between the comparator groups. If applicable, report incremental cost-effectiveness ratios. | Table 2  Table 3  Table 4 |
| Characterising uncertainty | 20a | *Single study-based economic evaluation:* Describe the effects of sampling uncertainty for the estimated incremental cost and incremental effectiveness parameters, together with the impact of methodological assumptions (such as discount rate, study perspective). | Figure 5;  Figure 6;  Figure 7;  Page 22, line 440 to 452;  Page 23, line 453 to 463 |
|  | 20b | *Model-based economic evaluation:*Describe the effects on the results of uncertainty for all input parameters, and uncertainty related to the structure of the model and assumptions. |  |
| Characterising heterogeneity | 21 | If applicable, report differences in costs, outcomes, or cost-effectiveness that can be explained by variations between subgroups of patients with different baseline characteristics or other observed variability in effects that are not reducible by more information. | Page 27, line 572 to 576;  Page 28, line 577 to 585 |
| **Discussion** | | | |
| Study findings, limitations, generalisability, and current knowledge | 22 | Summarise key study findings and describe how they support the conclusions reached. Discuss limitations and the generalisability of the findings and how the findings fit with current knowledge. | Page 23, line 470 to 476;  Page 24, line 477 to 501;  Page 25, line 502 to 506;  Page 26, line 527 to 551;  Page 27, line 552 to 571;  Page 28, line 586 to 600;  Page 29, line 601 to 604 |
| **Other** | | | |
| Source of funding | 23 | Describe how the study was funded and the role of the funder in the identification, design, conduct, and reporting of the analysis. Describe other non-monetary sources of support. | None (Page 1, line 25) |
| Conflicts of interest | 24 | Describe any potential for conflict of interest of study contributors in accordance with journal policy. In the absence of a journal policy, we recommend authors comply with International Committee of Medical Journal Editors recommendations. | None declared (Page 1, line 28) |
